# Do Hypertensive Individuals Who Are Aware of Their Disease Follow Lifestyle Recommendations Better than Those Who Are Not Aware?

**DOI:** 10.1371/journal.pone.0136858

**Published:** 2015-08-28

**Authors:** Yuna Kim, Kyoung Ae Kong

**Affiliations:** 1 Division of Health and Nutrition Survey, Korea Centers for Disease Control and Prevention, Cheongju-si, Chungcheongbuk-do, South Korea; 2 College of Nursing, Seoul National University, Seoul, South Korea; 3 Clinical Trial Center, Ewha Womans University Medical Center, Seoul, South Korea; The University of Tokyo, JAPAN

## Abstract

Lifestyle modification is the first step in hypertension management. Our objective was to assess adherence to lifestyle recommendations by individuals who were aware of their hypertension and to identify characteristics associated with non-adherence. Using data from the Korea National Health and Nutrition Examination Survey conducted in 2010–2012, we compared the adherence to six lifestyle recommendations of hypertensive subjects aware of the status of their condition with that of those who were not aware, based on survey regression analysis. The characteristics associated with non-adherence were assessed by multiple logistic regression analysis. Of all hypertensive subjects, <20% adhered to a healthy diet and reduced salt intake and about 80% moderated alcohol consumption and did not smoke. Half of all subjects maintained normal body weight and engaged in physical activity. Most lifestyle features of aware hypertensive Koreans did not differ greatly from those of hypertensive individuals who were not aware. Reduction in salt intake was slightly more prevalent among those aware of their hypertensive status. Obesity was more prevalent among the aware hypertensive subjects, and the prevalence of obesity increased with the duration of hypertension. Male gender, younger age, residence in a rural area, low income, and the use of antihypertensive medication were associated with non-adherence to lifestyle recommendations by hypertensive individuals. Many hypertensive Koreans do not comply with lifestyle recommendations for the management of hypertension. The association between the use of antihypertensive medications and non-adherence suggested an over-reliance on medication rather than a commitment to a healthy lifestyle. Our study highlights that efforts encouraging healthy lifestyles, as the first step in hypertension management, need to be increased.

## Introduction

Hypertension is a prime risk factor for cardiovascular disease, stroke, and chronic renal disease [1], and is the major contributor to the global disease burden [2]. Approximately 43,453 (15%) deaths and 670,707 (6.3%) disability-adjusted life years were attributable to high blood pressure (BP) in South Korea (hereinafter “Korea”) in 2010 [3].

Lowering BP can reduce the risk of diseases associated with hypertension [1, 4]. Although antihypertensive medication has been widely prescribed, lifestyle modification is also critical to the prevention and management of hypertension. The lifestyle modifications recommended by the Seventh Report of the Joint National Committee on Prevention, Detection, Evaluation, and Treatment of High Blood Pressure (JNC-7) are dietary sodium reduction, adoption of a Dietary Approaches to Stop Hypertension (DASH) eating plan, weight reduction, engagement in physical activity, moderation of alcohol consumption, and no smoking (to reduce the overall cardiovascular risk) [4]. Lifestyle modification reduces BP, enhances the efficacy of antihypertensive medication, and decreases cardiovascular risk. For example, limiting sodium intake upon the adoption of a DASH eating plan can reduce BP to a level similar to that attainable using one or two antihypertensive medications [4, 5]. Lifestyle modification should be the first step in hypertension treatment, and the need for a healthy lifestyle should be emphasized constantly for hypertension control. However, some hypertensive individuals rely principally on medication rather than on lifestyle changes and do not comply with lifestyle recommendations [4]. Furthermore, the lifestyle risk factors prior to the diagnosis of hypertension that contributed to the development of the disease render it difficult to assess adherence to the lifestyle recommendations for controlling hypertension. Thus, we sought to assess adherence to lifestyle recommendations for preventing and controlling hypertension among individuals who were aware of their hypertensive status and to compare it with that among those who were not aware. Moreover, we sought to identify characteristics associated with non-adherence to these recommendations.

## Methods

### Study population and data collection

This study was based on data obtained from the 5th Korea National Health and Nutrition Examination Survey (KNHANES) conducted in 2010–2012 by the Korea Centers for Disease Control and Prevention. This nationwide cross-sectional survey conducted every year features a complex, multi-stage clustered probability sampling design and a representative sample of the civilian, non-institutionalized South Korean population [6]. The survey consists of three distinct components: a health interview, a health examination, and a nutritional survey with food intake questionnaires. In 2011, the response rate was approximately 80%. Appropriate written informed consent for KNHANES was obtained from all participants, and patient records/information were anonymous and de-identified prior to analysis. We analyzed hypertensive subjects aged 40‒79 years drawn from the 5th KNHANES (2010–2012) dataset. Systolic and diastolic BP were the averages of the second and third readings of three measurements taken in a sitting position after at least 5 min of rest. Hypertension was defined as self-reported use of antihypertensive medication, a systolic BP ≥140 mmHg, or a diastolic BP ≥90 mmHg on health examination. Hypertensive subjects aware of their hypertension were defined as those diagnosed with hypertension by a doctor.

Of the total of 10,773 subjects aged 40–79 years who participated in the health interview, health examination, and nutrition survey, 4,440 had hypertension. We excluded 30 subjects who did not answer the question exploring a history of physician-diagnosed hypertension and 520 with physician-diagnosed renal failure, liver cirrhosis, stroke, myocardial infarction, or angina pectoris. A total of 88 subjects for whom data on current smoking status, alcohol intake, income, educational level, house ownership, physical activity level, or body mass index (BMI), was lacking, were also excluded. Finally, 3,802 subjects (1,625 males and 2,177 females) were included in the analysis. Of these, 2,786 subjects (73.2%) were aware of their disease and 1,016 were not.

### Lifestyle recommendations and other variables

From JNC-7, we used six operational definitions of adherence to lifestyle recommendations: salt intake reduction, adoption of a DASH-type diet, maintenance of a normal weight, engagement in physical activity, moderation of alcohol intake, and no smoking [1, 4]. We used information from the 24-hour recall nutritional survey to evaluate daily intake of vegetables/fruits and nutrients. Salt intake reduction was defined as dietary sodium consumption <2.3 g/day [5, 7]. We defined a DASH-type diet as a diet featuring (1) a potassium intake ≥4.7 g/day [1, 8]; (2) a total fat intake ≤25% of all calories [7, 9]; and (3) vegetable/fruit ingestion ≥500 g/day [10–12]. In terms of body weight, the JNC-7 guideline recommends a body-weight reduction of 4.5 kg, or ideally, the maintenance of normal body weight [4], but changes in body weight after the diagnosis of hypertension were not recorded in the survey. Thus, we defined the maintenance of a normal body weight as a BMI <25 kg/m^2^.

Health behaviors such as the physical activity level, alcohol consumption, and smoking status were explored in the health interview. Engagement in physical activity was defined as >30 min of walking or moderate physical activity ≥5 days a week or ≥20 min of vigorous physical activity ≥3 days a week [1, 4]. In terms of alcohol intake, we first calculated average daily alcohol consumption using data on the frequency of alcohol consumption and the average amount of alcohol consumed during each drinking occasion. Alcohol intake moderation was defined as ≤2 drinks/day in males and ≤1 in females [1, 4]. Not smoking was defined as not currently smoking [1, 4]. We summed the number of recommendations with which each subject complied.

The demographic and socioeconomic characteristics included sex, age, residential area (rural or urban), income (high, middle, or low), house ownership (no house or ownership of ≥1 house), and educational level (high school graduate or below, or college graduate or above). Residential areas were classified as urban (metropolitan cities and cities in the provinces) or rural based on government administrative classifications. Income quartile groups were based on sex- and age-specific quartiles of monthly-equivalent household income, which were calculated as monthly household income divided by the square root of the number of family members. We combined the second and third quartiles into a middle-income group. In terms of personal and family histories, we considered self-reported histories of doctor-diagnosed diabetes mellitus (DM), the time elapsed since the diagnosis of hypertension (<5, 5–9, or ≥10 years), the use of antihypertensive medication, family history of hypertension, and family history of ischemic heart disease (IHD) or stroke.

### Statistical analysis

In accordance with the complex sampling design, all analyses were performed incorporating the sampling weight, which accounts for unequal probabilities of selection, nonresponse, and the sex and age distributions in the target population (Korean Census 2005). Variances of all estimates were calculated using the Taylor linearization method.

We compared adherence to each recommendation by those who were aware of their hypertension with that by those who were not. The sex- and age-adjusted proportions of hypertensive subjects who adhered to each recommendation and the adjusted mean levels of nutrients or foods consumed among those aware and unaware of their hypertensive status were estimated based on age distributions among Koreans with hypertension. These were assumed to be 0.0875, 0.1281, 0.1575, 0.1400, 0.1410, 0.1405, 0.1176, and 0.0878 for the 5-year age groups from 40–44 to 75–79 years, respectively, and were also assumed to be the same for males and females. Between-group differences in terms of adherence to each recommendation, the mean levels of nutrients or foods consumed, and the number of recommendations with which subjects complied were analyzed using two survey regression models, one of which featured adjustment for sex and age, and the other adjustment for sex, age, residential area, family income, house ownership, education, history of DM, family histories of hypertension and stroke/IHD, and hypertension duration.

We also assessed characteristics associated with non-adherence to a greater number of lifestyle recommendations among subjects who were aware of their hypertension using an ordinal logistic regression analysis. Factors associated with non-adherence to each recommendation were also analyzed by multiple logistic regression analysis. All p-values refer to two-tailed tests, and p-values <0.05 were considered statistically significant. All statistical analyses were performed with SAS software (version 9.2, SAS Institute, Cary, NC). As the KNHANES dataset is publicly available, this study was exempt from institutional review board approval.

## Results

The characteristics of study subjects aware and not aware of their hypertensive status are shown in Table 1. Compared with subjects not aware of their hypertension, those subjects who were aware were older and more likely to be female. A higher proportion of aware respondents had DM and family histories of hypertension and stroke/IHD. Of the aware hypertensive subjects, 95.4% were taking antihypertensive medications, and their systolic and diastolic BP after adjustment for sex and age were 13.9 mmHg and 8.3 mmHg higher than those of the non-aware subjects (p<0.0001).

**Table 1:** The characteristics of hypertensive subjects who were and were not aware of their hypertension status.

Awareness of hypertension		Total (n = 3802)		Not aware (n = 1016)		Aware (n = 2786)		
		%	(SE)	%	(SE)	%	(SE)	p
Sex	Males	46.5	(0.8)	54.9	(1.5)	43.4	(0.9)	
	Females	53.5	(0.8)	45.1	(1.5)	56.6	(0.9)	
Age (year)	40–49	13.2	(0.7)	27.3	(1.6)	8.0	(0.6)	
	50–59	25.0	(0.8)	29.8	(1.5)	23.2	(0.9)	
	60–69	33.5	(0.8)	27.4	(1.5)	35.7	(1.0)	
	70–79	28.3	(0.8)	15.5	(1.1)	33.1	(1.0)	
Residence	Urban	73.1	(2.0)	74.9	(2.3)	72.4	(2.1)	0.6026
	Rural	26.9	(2.0)	25.1	(2.3)	27.6	(2.1)	
Income	Low	24.6	(0.9)	26.9	(1.5)	23.8	(0.9)	0.0547
	Middle	51.8	(1.0)	49.9	(1.6)	52.5	(1.1)	
	High	23.6	(0.9)	23.2	(1.5)	23.8	(1)	
House	No house	20.1	(0.8)	21.7	(1.4)	19.6	(0.9)	0.5054
	≥1 house	79.9	(0.8)	78.3	(1.4)	80.4	(0.9)	
Education	≤ High school	86.5	(0.7)	81.7	(1.4)	88.2	(0.7)	0.1186
	≥ College	13.5	(0.7)	18.3	(1.4)	11.8	(0.7)	
DM (doctor-diagnosed)	No	83.1	(0.6)	95.5	(0.7)	78.5	(0.8)	<0.0001
	Yes	16.9	(0.6)	4.5	(0.7)	21.5	(0.8)	
Family history of hypertension*	No	63.1	(0.9)	70.6	(1.4)	60.4	(1.1)	<0.0001
	Yes	36.9	(0.9)	29.4	(1.4)	39.6	(1.1)	
Family history of stroke or IHD*	No	80.3	(0.7)	81.1	(1.3)	80.1	(0.8)	0.0190
	Yes	19.7	(0.7)	18.9	(1.3)	19.9	(0.8)	
Time elapsed since diagnosis of hypertension (year)	Not diagnosed	26.8	(0.8)	100.0	(0.0)	.		
	<5	29.9	(0.8)	.		40.9	(1.0)	
	5–10	21.2	(0.7)	.		28.9	(1.0)	
	≥10	22.1	(0.8)	.		30.2	(1.0)	
Antihypertensive medication use	Not diagnosed	26.8	(0.8)	100.0	(0.0)	.		
	No	3.4	(0.3)	.		4.6	(0.4)	
	Yes	69.8	(0.9)	.		95.4	(0.4)	
Systolic blood pressure, mean (SE) (mmHg)	(crude)	135.4	(0.3)	144.4	(0.5)	132.1	(0.3)	<0.0001
	(adjusted***)	134.8	(0.3)	144.9	(0.4)	131.0	(0.4)	<0.0001
Diastolic blood pressure mean (SE) (mmHg)	(crude)	81.4	(0.2)	89.8	(0.3)	78.4	(0.2)	<0.0001
	(adjusted***)	82.8	(0.2)	88.9	(0.3)	80.6	(0.2)	<0.0001
Number of lifestyle recommendations**	0	1.6	(0.2)	2.1	(0.5)	1.4	(0.2)	0.3016
	1	6.4	(0.4)	7.7	(1.0)	5.9	(0.5)	
	2	25.2	(0.7)	27.9	(1.5)	24.2	(0.9)	
	3	37.1	(0.8)	35.3	(1.5)	37.7	(1.0)	
	4	24.2	(0.6)	22.9	(1.3)	24.6	(0.8)	
	5	5.6	(0.4)	4.1	(0.6)	6.1	(0.4)	
	6	0.0	(0.0)	0.0	(0.0)	0.1	(0.0)	

p-values adjusted for sex and age.

*father, mother, and siblings;

**recommendations were sodium intake reduction, adoption of a DASH-type diet, maintenance of normal weight, engagement in physical activity, moderation in alcohol intake, and not smoking. DM, diabetes mellitus; IHD, ischemic heart disease;

***sex- and age-adjusted mean.

Among all hypertensive subjects, the recommendation with the lowest level of adherence was adoption of a DASH-type diet (10.6%); among its components, adherence to a high level of potassium intake was the lowest (Table 2). The next lowest level of compliance was garnered by the recommendation to reduce salt intake (17.8%). The highest proportions of subjects complied with the recommendations of moderate alcohol consumption and not to smoke, both of which had adherence levels of approximately 80%. Half the hypertensive respondents maintained a normal body weight and engaged in physical activities.

**Table 2:** Adherence to lifestyle recommendations among hypertensive subjects by awareness of hypertensive status.

Awareness of hypertension			Not aware (n = 1016)		Aware (n = 2786)					
	Adherence* (%)	(SE)	Adherence* (%)	(SE)	Adherence* (%)	(SE)	p*	β**	(SE)	P**
Salt intake reduction (Na ≤2.3g/d)	17.8	(0.6)	15.6	(1.1)	18.6	(0.8)	0.0252	3.8	1.5	0.0141
Adoption of a DASH-type diet	10.6	(0.6)	11.0	(1.1)	10.5	(0.7)	0.6621	-2.1	1.4	0.1244
Fat ≤25% of total energy intake	88.5	(0.6)	89.5	(1.0)	88.2	(0.8)	0.2701	-1.2	1.3	0.3629
K intake ≥4.7 g/d	14.1	(0.7)	13.9	(1.2)	14.1	(0.8)	0.8774	-1.1	1.5	0.4544
Vegetable/fruit intake ≥500 g/d	45.6	(1.0)	43.4	(1.6)	46.4	(1.2)	0.1231	1.7	2.2	0.4464
Maintenance of normal weight (BMI<25 kg/m^2^)	48.3	(0.9)	55.8	(1.5)	45.5	(1.1)	0.0000	-6.7	2.1	0.0016
Engage in physical activity	46.4	(1.0)	47.3	(1.7)	46.1	(1.1)	0.5486	-1.7	2.2	0.4378
Moderation of alcohol intake	81.5	(0.7)	78.9	(1.3)	82.5	(0.8)	0.0152	2.2	1.6	0.1662
Not smoking	82.1	(0.7)	80.3	(1.2)	82.7	(0.8)	0.1010	2.2	1.7	0.1994
Number of recommendations complied with										
≥ 1	98.1	(0.3)	98.5	(0.4)	97.9	(0.3)	0.1984	-0.3	0.6	0.6344
≥ 2	90.8	(0.6)	92.0	(0.9)	90.3	(0.7)	0.1122	-2	1.2	0.1016
≥ 3	64.4	(0.9)	64.9	(1.5)	64.3	(1.0)	0.7149	0.3	2	0.8652
≥ 4	28.1	(0.7)	28.9	(1.4)	27.8	(0.9)	0.5065	-1.6	1.9	0.3933
≥ 5	5.3	(0.4)	4.5	(0.6)	5.6	(0.5)	0.1505	1.3	0.9	0.1503
	Mean*	(SE)	Mean*	(SE)	Mean*	(SE)	p*	β**	(SE)	P**
Energy intake (kcal/d)	1991.5	(15.7)	2007.3	(25.5)	1985.6	(18.1)	0.4586	-15.8	(32.6)	0.6275
Na intake (mg/d)	4980.9	(60.0)	5073.6	(110.7)	4946.9	(67.0)	0.3040	-197.6	(140.0)	0.1586
Fat % of total energy intake	15.2	(0.2)	15.0	(0.3)	15.2	(0.2)	0.4259	0.2	(0.3)	0.5930
Potassium intake (mg/d)	3137.8	(32.5)	3082.0	(50.2)	3158.3	(37.5)	0.1862	-20.7	(65.7)	0.7534
Vegetable/fruit intake (g/d)	554.7	(8.1)	555.0	(13.9)	554.6	(9.3)	0.9815	-20.9	(18.0)	0.2441
BMI (kg/m^2^)	25.4	(0.1)	24.7	(0.1)	25.6	(0.1)	0.0000	0.6	(0.1)	0.0001
Alcohol intake (drinks/day)	1.00	(0.03)	1.14	(0.06)	0.95	(0.04)	0.0070	-0.14	(0.08)	0.0795
Number of recommendations complied with	2.87	(0.02)	2.89	(0.03)	2.86	(0.02)	0.4274	-0.02	(0.04)	0.5909

Engage in physical activity: >30 min of walking or moderate physical activity ≥5 days a week or ≥20 min of vigorous physical activity ≥3 days a week; moderation of alcohol intake: ≤2 drinks/day in males and ≤1 in females.

*Adherence adjusted for sex and age via survey regression analysis;

**β: mean difference adjusted for sex, age, residential area, educational level, income, and house ownership, history of diabetes mellitus, family histories of hypertension and stroke/ischemic heart disease, and duration of hypertension compared with those who were not aware of their hypertension status.

Members of both groups (aware of hypertensive status or not) complied with an average of less than three lifestyle recommendations (Table 2), and adherence rates were not different between the groups, when using any of the recommendation cutoff values (Tables 1 and 2). Compared with non-aware hypertensive subjects, 3 percentage points (%p) more aware subjects limited their salt intake to <2.3 g/day after adjustment for sex and age (Table 1, p = 0.0252). However, the means of daily salt consumption were 4,947 mg and 5,074 mg in those who were and were not aware, respectively; these values did not differ significantly and are much higher than the recommended level. The sex- and age-adjusted proportions of adherence to the recommendations of moderate alcohol intake and no smoking among those aware of their hypertensive status were 3.6%p and 2.4%p higher, respectively, than those among participants who were not aware. After adjustment for sex, age, and other covariates, the differences did not attain statistical significance. Compliance with a DASH-type diet and engagement in physical activity did not differ by awareness of hypertension. Mean potassium and vegetable/fruit consumption and the percentage of fat of total energy intake also did not significantly differ between the two groups. Aware hypertensive subjects were significantly more obese than those who were not aware; the mean BMI of the former group was 0.6 kg/m^2^ higher than that of the latter group after full adjustment. Maintenance of normal body weight was much less common among the aware (45.5%) compared with the non-aware (55.8%), and this difference was 6.7%p after full adjustment (p = 0.0016).

Ordinal logistic regression analysis of the factors associated with the number of recommendations to which respondents did not adhere revealed that hypertensive males were two-fold more likely to be non-adherent than were females (OR 1.98, 95% CI 1.70–2.32), and that younger hypertensive individuals were less likely to comply with the recommendations (Table 3). Rural residence and low income were associated with non-adherence to a greater number of recommendations, with ORs of approximately 1.3. Hypertensive subjects taking antihypertensive medications had higher odds of non-adherence (OR 1.64, 95% CI 1.15–2.35) than those who did not take medications.

**Table 3:** Characteristics associated with non-adherence among hypertensive subjects aware of their hypertensive status.

			Non-adherence to a greater number of recommendations			Not limiting Sodium intake			Not adopting a DASH-type diet			Not maintaining a normal body weight			Not engaging in physical activity			Not limiting alcohol intake			Smoking		
		N	%*	OR**	(95% CI)	%	OR	(95% CI)	%	OR	(95% CI)	%	OR	(95% CI)	%	OR	(95% CI)	%	OR	(95% CI)	%	OR	(95% CI)
Sex	Females	1676	23.9	1		70.6	1		93.3	1		53.7	1		57.2	1		4.4	1		3.2	1	
	Males	1110	41.3	1.98	(1.70–2.32)	88.1	2.85	(2.32–3.50)	88.0	0.57	(0.43–0.75)	49.7	0.82	(0.70–0.96)	50.0	0.76	(0.64–0.89)	26.4	8.02	(6.13–10.51)	27.7	13.25	(9.87–17.79)
Age (year)	40–49	201	48.8	0.97	(0.96–0.98)	89.2	0.96	(0.95–0.97)	86.0	1.05	(1.04–1.07)	64.9	0.97	(0.96–0.98)	62.1	1.00	(0.99–1.01)	22.7	0.97	(0.95–0.98)	24.8	0.96	(0.94–0.97)
	50–59	623	35.6			83.4			86.7			53.4			54.1			17.5			18.6		
	60–69	990	30.8			80.0			90.7			53.1			50.4			13.9			12.4		
	70–79	972	25.1			69.9			95.5			46.6			56.1			9.3			9.4		
Residence	Urban	2002	34.1	1		81.9	1		88.4	1		53.5	1		53.6	1		16.0	1		16.5		
	Rural	784	41.5	1.30	(1.11–1.54)	81.8	1.04	(0.84–1.29)	92.1	1.54	(1.05–2.26)	55.8	1.12	(0.93–1.35)	58.7	1.17	(0.96–1.44)	20.5	1.49	(1.11–2.00)	20.4	1.34	(1.02–1.76)
Income	High	653	32.3	1		84.0	1		86.3	1		52.9	1		48.9	1		19.1	1		13.5	1	
	Middle	1463	36.4	1.20	(1.00–1.43)	81.6	0.92	(0.72–1.17)	89.1	1.31	(0.95–1.81)	53.8	1.01	(0.83–1.24)	55.9	1.30	(1.09–1.55)	16.9	0.75	(0.56–1.00)	17.8	1.42	(1.02–1.97)
	Low	670	38.7	1.37	(1.10–1.70)	80.0	0.87	(0.65–1.16)	92.9	2.26	(1.35–3.77)	56.0	1.11	(0.88–1.41)	58.9	1.44	(1.15–1.80)	15.9	0.63	(0.44–0.91)	20.6	1.70	(1.15–2.51)
House	≥ 1 house	2231	35.2	1		82.4	1		88.7	1		53.6	1		54.7	1		16.8	1		16.6	1	
	No house	555	39.2	1.18	(0.99–1.41)	79.5	0.87	(0.68–1.11)	92.0	1.57	(1.01–2.42)	56.2	1.12	(0.91–1.37)	55.9	1.07	(0.88–1.30)	18.5	1.27	(0.93–1.75)	21.0	1.47	(1.07–2.03)
Education	≥ college	298	27.9	1		85.9	1		87.1	1		53.9	1		49.8	1		14.2	1		7.7	1	
	≤ high school	2488	37.6	1.17	(0.92–1.49)	81.0	0.70	(0.47–1.06)	89.8	0.91	(0.61–1.35)	54.1	0.92	(0.72–1.19)	56.0	1.06	(0.81–1.40)	17.8	1.28	(0.89–1.85)	19.4	1.91	(1.33–2.75)
DM (doctor-diagnosed)	No	2202	35.4	1		81.8	1		89.7	1		52.8	1		55.1	1		17.2	1		16.8	1	
	Yes	584	38.4	1.17	(0.97–1.39)	81.9	1.02	(0.81–1.29)	87.7	0.75	(0.54–1.04)	59.5	1.22	(1.02–1.47)	54.4	0.98	(0.81–1.18)	17.1	1.04	(0.78–1.38)	20.3	1.45	(1.07–1.97)
Family history of hypertension	No	1700	35.3	1		80.6	1		89.4	1		55.0	1		55.0	1		17.2	1		17.6	1	
	Yes	1086	36.8	1.03	(0.87–1.21)	83.3	1.13	(0.92–1.39)	89.3	1.14	(0.83–1.57)	53.1	0.87	(0.73–1.05)	54.9	1.03	(0.86–1.22)	17.1	1.08	(0.83–1.40)	17.3	1.11	(0.83–1.49)
Family history of stroke or IHD	No	2238	36.1	1		81.4	1		89.8	1		53.7	1		54.6	1		17.7	1		17.8	1	
	Yes	548	35.6	0.99	(0.82–1.19)	83.6	1.08	(0.85–1.38)	87.5	0.82	(0.58–1.16)	55.7	1.09	(0.89–1.33)	56.1	1.06	(0.86–1.31)	15.3	0.78	(0.59–1.02)	16.3	0.92	(0.66–1.28)
Duration since diagnosis of HTN (years)	<5	1140	36.7	1		82.4	1		90.9	1		49.9	1		55.8	1		19.5	1		18.8	1	
	5–10	806	33.9	0.86	(0.72–1.02)	79.9	0.85	(0.68–1.07)	89.7	0.77	(0.54–1.10)	54.6	1.19	(0.99–1.44)	52.6	0.85	(0.70–1.04)	16.6	0.78	(0.59–1.02)	16.8	0.77	(0.57–1.06)
	≥10	840	37.1	0.97	(0.81–1.15)	83.0	1.00	(0.78–1.27)	86.4	0.55	(0.40–0.77)	60.4	1.52	(1.24–1.85)	56.0	1.01	(0.82–1.23)	14.1	0.61	(0.44–0.86)	16.1	0.79	(0.57–1.09)
Antihypertensive medication use	No	130	27.2	1		77.5	1		84.0	1		38.7	1		53.2	1		18.3	1		18.2	1	
	Yes	2656	36.6	1.64	(1.15–2.35)	82.1	1.48	(0.94–2.33)	89.7	1.65	(0.94–2.91)	55.2	1.81	(1.25–2.63)	55.0	1.01	(0.70–1.45)	17.1	0.91	(0.56–1.50)	17.4	0.88	(0.53–1.44)

All proportions (%) were sex- and age-adjusted.

*Proportion of non-adherence to ≥4 recommendations;

**Ordinal logistic regression for non-adherence to a greater number of recommendations.

The patterns of associations involving non-adherence to each lifestyle recommendation were generally similar to those involving non-adherence to a greater number of recommendations, but some differences were also apparent. Females were less likely to adopt DASH-type diets, to maintain a normal body weight, and to engage in physical activity. Older hypertensive individuals were more likely to not adopt a DASH-type diet. High income was associated with non-limitation of alcohol consumption. Subjects with DM were less likely to attain a normal body weight (OR for not maintaining a normal weight 1.22, 95% CI 1.02–1.47) and to stop smoking (OR for smoking 1.45, 95% CI 1.07–1.97). Those who had hypertension of longer duration were more likely to adopt DASH-type diets and to limit alcohol intake but were less likely to attain normal body weight (OR for ≥10 years compared with <5 years 1.52, 95% CI 1.24–1.85). Subjects who took antihypertensive medication were less likely to attain normal body weight (OR 1.81, 95% CI 1.25–2.63) and (although not statistically significant) were more likely not to limit sodium intake or adopt a DASH-type diet (OR 1.48, 95% CI 0.94–2.33, p = 0.088 and OR 1.65, 95% CI 0.94–2.91, p = 0.081, respectively)

## Discussion

Adoption of a healthy lifestyle is important in the management of hypertension [4], but our present study, using data from a nationally representative survey, showed that most lifestyle features of hypertensive Koreans aware of their condition were similar to those who were not aware. The level of adherence to salt intake reduction was slightly higher among those aware of their hypertension, but maintenance of normal body weight was considerably less common. The average number of lifestyle recommendations with which both aware and unaware hypertensive subjects complied was fewer than three of the six evaluated. Male gender, relative youth, living in a rural area, low income, and use of antihypertensive medication were generally associated with non-adherence to recommendations among aware hypertensive individuals.

The phenomenon of a low level of adherence by hypertensive individuals to lifestyle recommendations is not unique to Korea. Although some studies reported that a substantial proportion of hypertensive individuals did change their health behaviors and adopt healthy lifestyles [13–15], these findings were based on self-reported compliance, and it is well known that such data overestimate actual adherence [13]. Two European studies that compared adherence between aware and unaware hypertensive subjects concluded that more aware subjects adhered to recommendations such as not smoking or limiting alcohol or sodium intake, but the differences were small, and there were no differences for other recommendations [16, 17]. In a study of a Dutch population conducted in the 1990s, the number of guidelines the aware subjects met was slightly greater than the unaware subjects did (5.0 and 4.8 of 10 evaluated), and approximately 7%p more adhered to the guidelines for maintaining a BMI <27 kg/m^2^ and consuming <20g/d alcohol among those aware than those unaware [17]. However, a more recent study conducted in Spain showed that adherence to not smoking was 11%p higher in the aware than the unaware hypertensive subjects but 6.4%p lower for maintaining a BMI <25 kg/m^2^, and there was no substantial difference in adherence to physical activity [16]. These findings were more similar to our results for adherence to a normal BMI and physical activity. In fact, our ultimate concern was the lifestyle changes made among hypertensive subjects, although the cross-sectional design of this study could not resolve this. To the best of our knowledge, the only longitudinal population study reported few lifestyle changes 2 years after the first diagnosis of hypertension in Canadian patients [18]; improvements related to not smoking and engaging in physical activity were found in only 5%.

The rate at which aware hypertensive subjects limited salt intake was higher than that among non-aware ones, but adherence by 18.6% and a mean salt intake of 4,947 mg/d were far from optimal. The reason may be traditional Korean diets, which feature kimchi (a sort of pickled cabbage), soy sauce, and soybean paste [19]. However, this non-optimal level of sodium intake has also been observed in both hypertensive and general populations worldwide [20–23]. The proportions of hypertensive subjects consuming <2.3 g/d Na were less than 15% in both the USA and China [21, 22]. Recently, reducing sodium intake has become a significant public health issue [5, 20, 23], and this topic is complex, involving multiple stakeholders [5]. Those with hypertension and other cardiovascular risk factors should be the primary targets of the efforts to promote reduction in sodium intake. Such individuals could be prioritized in terms of education and consultation under medical care. Salt intake in Korea principally comes from home-processed foods or condiments added during cooking, which differs from the situation in Western countries [19, 20]. Thus, any change in salt intake among Koreans would be individualistic in nature; thus, education and consultation may facilitate more rapid changes than are possible in other countries in which processed or restaurant-prepared foods are the principal sources of sodium.

The low level of adherence to a DASH-type diet was attributed to low levels of adherence to increased intake of dietary potassium and diets rich in vegetables/fruits, rather than to a low-fat diet. Fewer than 15% of hypertensive individuals consumed ≥4.7 g/d potassium; the mean potassium intake was, in fact, slightly less than 3.2 g/d, which is similar to the mean of the Korean general population (approximately 3 g/d) [19]. This level was slightly lower than those of European countries, which have a mean potassium intake of 3.1–4.8 g/d [24, 25], and slightly higher than those of the U.S., Japan, and China [21, 22, 26]. Potassium intake in all surveyed countries is suboptimal. Potassium intake should be increased by the consumption of fruits/vegetables and nuts, but supplements are not generally advised [1, 8]. Adherence to such dietary regimens is challenging, because low levels of consumption of fruits/vegetables are generally closely associated with socioeconomic status (SES) [27–30]. Low SES is related to lower adherence to dietary recommendations and to lower diet quality and diversity, as well as lower intake of fruits/vegetables [30]. As found elsewhere, low income, the absence of house ownership, and residence in a rural area mitigated against adoption of a DASH-type diet in our study. Strategies designed to increase potassium intake should reduce the fruit/vegetable consumption gap across SES, as well as emphasize the need to increase potassium intake, identifying suitable foods.

Hypertensive individuals aware of their hypertension were more obese than were those who were unaware; the proportion of obese subjects in the former group was about 10%p higher than in the latter. Obesity may have been relevant prior to diagnosis and the associated awareness of hypertension, and the probability that hypertension may be diagnosed likely increases in obese individuals. However, even among those aware of their hypertension, individuals with hypertension for ≥5 years exhibited more than 5%p higher prevalence of obesity compared with those whose hypertension was of shorter duration. Furthermore, hypertensive individuals with comorbid conditions, such as DM, were less likely to attain normal body weights. An increase in obesity among hypertensive patients has ever been reported. According to a Canadian longitudinal population study, the proportion of obese subjects significantly increased (by 2.8%p) 2 years after the initial diagnosis of hypertension [18]. The increase was more substantial among those who took antihypertensive medications, which is consistent with our finding that the use of such medications was associated with increased odds for non-adherence to maintenance of a normal body weight and to a greater number of recommendations. Neutel et al. sought to explain such results by reference to the mechanisms of beta-blockers [18]. We suggest that many hypertensive patients believe that antihypertensive medication is sufficient to control their hypertension, and thus did not accept that lifestyle modifications, including maintenance of a normal body weight, were essential components of hypertension management [4]. The use of antihypertensive medications was also associated with increased odds of non-adherence to several recommendations: reduced salt intake and adoption of a DASH-type diet (although the latter association was not statistically significant). These data support our interpretation. Over-reliance on antihypertensive medications has been suggested in several studies [4, 18, 31], and such a tendency would be stronger when a national health insurance scheme or service covers the costs of such medications.

Approximately half of all hypertensive individuals engaged in physical activity, and 80% of them limited their alcohol intake and did not smoke, but no substantial between-group difference in terms of hypertension awareness was evident. The levels of adherence that we noted were similar to those of hypertensive patients in the USA, Canada, China, and Israel [13–15, 18, 32]. However, the extent of engagement in physical activity by Korean hypertensive individuals was also similar to that of normotensives [33]. Furthermore, hypertensive individuals with DM were more likely to smoke than were those without DM. Higher-income subjects were less likely to limit alcohol intake, reflecting an aspect of Korean culture: alcohol consumption is generally accepted as an important component of business and social relationships [34]. The low compliance of these unexpected subgroups in our study underscored the need to assess the adequacy of education or counseling programs promoting the adoption of healthy lifestyles, as well as the knowledge regarding and attitudes toward disease, medication, and health-related behavioral recommendations. Longitudinal lifestyle changes among hypertensive subjects also require attention. On the other hand, indicators of low SES such as low income and no house ownership were associated with low levels of adherence to not smoking and engaging in physical activity, as well as to adopting a DASH-type diet. These findings were the same as those of a study conducted in the general Korean population [34]. Poor living conditions with less available or accessible facilities and green surroundings and poor coping strategies associated with psychological stress and less health-related knowledge can hamper the adoption of healthy lifestyles, and are a reason for the higher incidence of cardiovascular disease in low-SES populations [7, 34]. More efforts toward health policies, education/counseling, and targeted strategies to reduce socioeconomic disparities in adopting healthy behaviors are needed for optimal hypertension management.

This study has several limitations. First, the cross-sectional design does not allow the identification of temporal and causal relationships between lifestyle and hypertension awareness [35]. Obesity may encourage subjects to seek medical attention, thereby facilitating the early diagnosis of hypertension. However, although we assume that obesity that develops early in the course of hypertension contributes to awareness of hypertension, the between-group differences in BMI and obesity prevalence by hypertension duration (over 5%p) are attributable to lifestyle changes made after the diagnosis of hypertension. In addition, because our principal objective was to explore the extent to which recommended lifestyles were followed by hypertensive individuals who might already have risk factors for this condition (e.g., an unhealthy diet, a sedentary lifestyle, or obesity), we chose hypertensive subjects who were not aware of their hypertension as the most appropriate controls. Thus, we show how diagnosis and awareness of personal hypertension affected the health behavior of and adherence to recommendations in Korean hypertensive individuals, although our work was not longitudinal in nature. Second, most health behaviors were assessed subjectively, including via 24-h dietary recall. Adherence to lifestyle recommendations measured by self-report generally tends to be overestimated [13], and over/underestimation or differential reporting by disease or SES status may have affected results [13, 21, 32, 36]. Third, the operational definition for a DASH-type diet did not include information about the consumption levels of low-fat dairy products, saturated fat, cholesterol, or calcium due to limited information available in the survey and nutrient database. Thus, we may have overestimated adherence to a DASH-type diet. Despite these limitations, the use of nationally representative survey data and the formation of an appropriate comparison group allowed us to assess adherence to lifestyle recommendations among the hypertensive population. To the best of our knowledge, this is the first nationwide population-based study on adherence to such recommendations in Asian hypertensive individuals. Furthermore, as KNHANES did not explore changes in health behaviors or compliance with recommendations but rather examined the existence of each health behavior per se, estimates of adherence would be much less overestimated in the present study than in studies employing other questionnaires. Thus, we believe our findings can contribute to hypertension management from a public health perspective.

In conclusion, many hypertensive Koreans did not comply with the lifestyle recommendations offered to help with the management of hypertension, especially with regard to the adoption of a DASH-type diet, limiting salt intake, and maintaining a normal weight. The adherence of those who were aware of their hypertension was higher than that of those lacking awareness only in terms of limiting sodium intake, and this difference was only <5%p. Obesity was more prevalent and increased with the duration of hypertension among aware hypertensive individuals. Lifestyle modification is the first step in the effective treatment of hypertension and can both avoid the adverse effects of medication and reduce individual and public costs [18]. This study highlights that adherence to lifestyle recommendations by those with hypertension is low and emphasizes the need for improved lifestyle counseling and education, including more effective strategies and aggressive approaches, to help hypertensive Koreans lead a healthy lifestyle.
